# Dual modality prompt learning for visual question-grounded answering in robotic surgery

**DOI:** 10.1186/s42492-024-00160-z

**Published:** 2024-04-22

**Authors:** Yue Zhang, Wanshu Fan, Peixi Peng, Xin Yang, Dongsheng Zhou, Xiaopeng Wei

**Affiliations:** 1 National and Local Joint Engineering Laboratory of Computer Aided Design, School of Software Engineering, Dalian University, Dalian, 116622 Liaoning China; 2 School of Computer Science and Technology, Dalian University of Technology, Dalian, 116081 Liaoning China

**Keywords:** Prompt learning, Visual prompt, Textual prompt, Grounding-answering, Visual question answering

## Abstract

With recent advancements in robotic surgery, notable strides have been made in visual question answering (VQA). Existing VQA systems typically generate textual answers to questions but fail to indicate the location of the relevant content within the image. This limitation restricts the interpretative capacity of the VQA models and their ability to explore specific image regions. To address this issue, this study proposes a grounded VQA model for robotic surgery, capable of localizing a specific region during answer prediction. Drawing inspiration from prompt learning in language models, a dual-modality prompt model was developed to enhance precise multimodal information interactions. Specifically, two complementary prompters were introduced to effectively integrate visual and textual prompts into the encoding process of the model. A visual complementary prompter merges visual prompt knowledge with visual information features to guide accurate localization. The textual complementary prompter aligns visual information with textual prompt knowledge and textual information, guiding textual information towards a more accurate inference of the answer. Additionally, a multiple iterative fusion strategy was adopted for comprehensive answer reasoning, to ensure high-quality generation of textual and grounded answers. The experimental results validate the effectiveness of the model, demonstrating its superiority over existing methods on the EndoVis-18 and EndoVis-17 datasets.

## Introduction

Visual question answering (VQA) has emerged as a pivotal multimodal task in recent years, seamlessly integrating visual and language components. With the development of deep learning, the VQA system has the potential to serve as an auxiliary tool with extensive applications in the healthcare domain, offering valuable assistance to physicians in diagnosis and decision-making [1–3]. Most existing deep-learning-based VQA models primarily generate and validate these textual responses [4–6]. However, such a validation mechanism is too simple to ensure that the model correctly answers questions based on visual content. Consequently, research efforts have been dedicated to enhancing answer validation mechanisms for a more reliable VQA system [7–10].

Recently, some datasets in the field of VQA have incorporated a grounded answer validation mechanism [11–13]. This verification process involves the accurate identification of specific regions in the image corresponding to the textual answers, thereby enhancing both the accuracy (ACC) and interpretability of the answers. These developments have increased the feasibility of developing secure and reliable VQA models for medical applications. Subsequently, some works [14–16] introduced visual question-grounded answering (VQGA) specifically for robotic surgery, to establish a correspondence between the answers and the spatial location of objects within the surgical scene. Bai et al. [15] proposed the co-attention gated vision-language embedding** (**CAT-ViL) and surgical*-*visual question localized-answering (surgical-VQLA) [14] models to enhance the embedding of multimodal information. This enhancement facilitates the generation of text and corresponding foundational answers, thereby enabling a better understanding of the surgical scene. Similarly, the CS-VQLA [16] model attempts to address the problem of continuous model learning in complex surgical scenes through distillation, providing more accurate and localized answers.

Although these models effectively integrate problems and image information to improve system ACC, their dependence on the visual and textual prompts from the current surgical scenario hampers their ability to comprehensively address diverse instances within the same surgical context in answering relevant questions. Consequently, augmenting the VQGA model’s understanding of global knowledge is imperative for refining the precise localization of local information. This refinement is crucial for meeting the stringent demands of real-time decision-making and ACC in surgical operations.

In this study, we propose a framework that combines prompt learning and pre-training models for VQGA in robotic surgery, enabling the model to attend to global information based on the prompted knowledge. To better utilize the knowledge in the prompts and supplement the visual and semantic information of each image, we designed a cross-modal prompt interaction mechanism that integrates the prompted knowledge into the encoding end of the CAT-ViL [15] model through cross attention, focusing the model attention on capturing fine-grained information for subsequent matching instances, grounding the answers on images and text. Specifically, two simple and lightweight prompt fusion modules were proposed to insert into the encoding end of the base model. The visual complementary prompter (VCP) integrates visual box prompt features with the original image encoding, whereas the text complementary prompter (TCP) module fuses visual box prompt features with the original question encoding and labels the features of the prompt. This combination allows the model to absorb rich visual and semantic information by prompting knowledge at the encoding end. Notably, an iterative fusion strategy was adopted to hierarchically superimpose and interact with different information, aiming to promote better alignment and fusion between different modal and question prompt features. The contributions of this study are summarized as follows:

1. A VQGA framework based on dual-modality prompt learning is proposed to effectively explore the prompted knowledge in robotic surgery tasks for better grounded answer generation.
2. Two prompters are proposed to complement visual and textual information, whereby a multiple iterative fusion strategy is adopted to encode the knowledge in prompts and multimodal features, which facilitates the aggregation of complementary information across multiple feature levels.
3. Using the proposed model, state-of-the-art performance was obtained on two challenging datasets, surpassing the achievements of previous studies by a considerable margin.

Explainable VQA systems have proven to be reliable in the medical field [9, 11, 12]. With the medical artificial intelligence field flourishing [7, 17], VQA systems based on robotic surgery have been extensively developed and applied. VQA systems can answer questions about specific visual elements in surgical videos or images [8, 16]. For example, surgeons can ask the system questions about certain aspects of the surgical field, such as identifying specific tissues, organs, and surgical tools. However, a key problem with robotic surgery-based VQA systems is their lack of interpretability. Although these systems can provide textual answers to questions, they cannot highlight the relevant regions of the image corresponding to the textual answers. Surgical scenarios often involve various instruments and actions that can confuse the questioner. To help questioners deal with this confusion, researchers [14, 17] proposed the establishment of a VQGA system to effectively learn and understand surgical scenes.

The surgical-VQLA [14] model combines a visual transformer with a gating visual-linguistic embedding system to accurately locate specific surgical areas during answer prediction. The CAT-ViL model proposed by Bai et al. [15] achieves answer grounding by emphasizing the effective integration of multimodal inputs. The latest CS-VQLA [16] model utilizes distillation to achieve continuous learning, resulting in more accurate textual and grounded answers. Both studies emphasize the importance of integrating visual and language data in robotic surgery to improve the ACC and efficiency of answers. However, to enhance their practical applicability, these models must achieve higher ACC levels and utilize the rich visual and semantic information inherent in the images and text more effectively.

In recent years, there has been rapid development in the field of multimodal pre-training of large-scale models. Researchers often use fine-tuning to leverage pretrained large models for downstream tasks. This method is often inefficient in terms of parameters, usually requiring numerous copies specific to each task and substantial storage for each version of the fully pretrained model. Recently, the emergence of prompt learning as a new paradigm has drastically improved the performance of various downstream natural language processing tasks [18, 19], and has been effective in several computer vision tasks. For example, the Visual Prompt Tuning model proposed by Jia et al. [20] adds a set of learnable parameters to transformer encoders and has been demonstrated superior to complete fine-tuning in 20 downstream recognition tasks. The AdaptFormer network introduced by Chen et al. [21] integrates lightweight modules into the vision transformer, achieving better results than fully fine-tuned models in action recognition benchmarks. The convolutional bypass model proposed by Jie and Deng [22] utilizes convolutional bypasses in pretrained vision transformers for prompt learning. The designs of these prompts often strategically utilize prior knowledge and capabilities of the model to direct attention or provide explanations for the expected results. The effectiveness of prompt learning largely depends on the architecture of the underlying model and training on relevant datasets. Inspired by this, we introduced prompt learning into the VQGA system for further exploration, incorporating dual-modality prompt knowledge from both visual and textual sources. A novel prompt-learning framework specifically tailored for VQGA was developed to better utilize the potential knowledge contained in each modality prompt. To the best of our knowledge, this is the first study to apply prompt learning to VQGA systems for robotic surgery.

## Methods

### Architectural overview

**Fig. 1:**
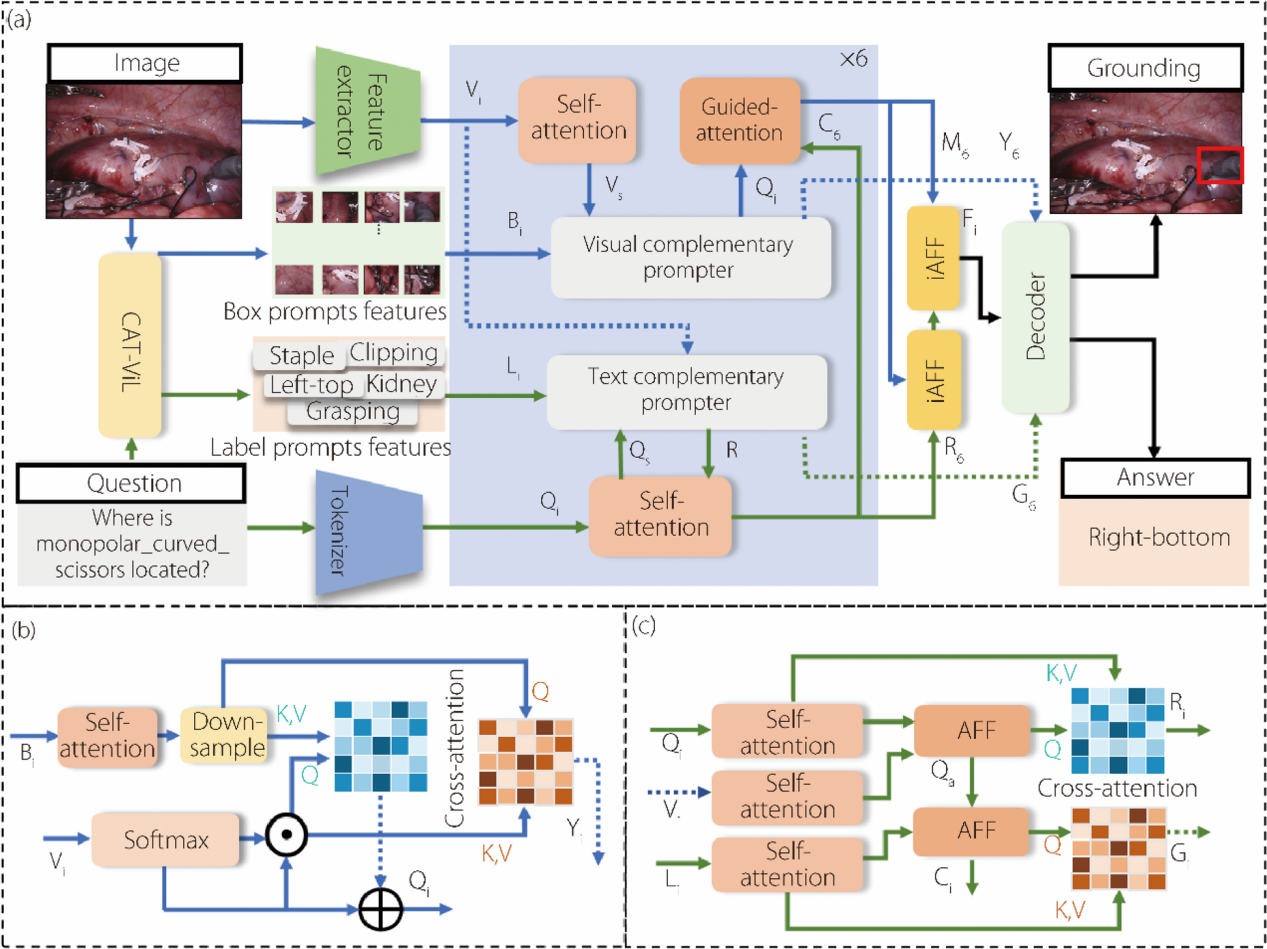
Overall architecture of the proposed DMPL. **a** Dual modality prompt learning (DMPL) network; **b** VCP; **c** TCP

As shown in Fig. 1a, we propose a VQGA framework based on bimodal prompts. Initially, the framework utilizes the pretrained CAT-ViL [15] model to extract prompts for visual and textual knowledge, referred to as box and label prompt features, respectively. CAT-ViL is a model trained for the VQLA task and can directly obtain prompt features by configuration. The questions and images are input into the model, whereby the model outputs information on three localized visual features and corresponding three label features. These serve as the prompt features in our model. To better utilize the prompt knowledge from both modalities, we designed complementary visual and text prompters. These are integrated with the existing pretrained model through a layered iterative fusion approach using the prompt information to guide the interaction and fusion of multimodal information.

The overall architecture of DMPL for VQGA in robotic surgery is presented in Fig. 1. The proposed network components include the VCP and TCP. The pretrained CAT-ViL model [15] is leveraged to extract prompt features for visual box prompt and textual label prompt features. The VCP integrates visual prompt and visual features through a cross-attention mechanism, aiming to guide the localization of visual features using the features of visual prompts. TCP aligns questions and visual features using the attentional feature fusion (AFF) module [23], subsequently aligning these with label prompt features to synchronize the three types of information before guiding them through cross-attention. The goal is to generate the correct answer by jointly guiding textual information with label prompt and visual features. Six fusion iterations are performed between the prompt features and extracted original features, as indicated by the blue box in Fig. 1a, to ensure more effective guidance. Subsequently, the refined visual and textual data obtained through complex reasoning are merged using the stacked iterative attentional feature fusion (iAFF) module [23]. The combined data are then decoded along with the post-reasoning visual and textual prompt features. Finally, the model generates the final textual answers and grounded answers through a dedicated classification head and an object detection head.

To enhance the utilization of these bimodal prompt systems, separate generators were designed for the visual and textual supplementary prompts. These generators were integrated with the original pretrained model using a layered iterative fusion approach. This method employs prompts to direct the interaction and fusion of multimodal information, thereby enabling more precise generation of textual answers and localization of visually grounded answers. The proposed architecture also enhances the utilization of local visual regions. The TCP incorporates the features of visual regions and aligns them with both textual and text prompt features. This alignment guides the textual information through visual information, thereby mitigating the language bias in VQA models to generate textual answers through reasoning based on visual information. To strengthen the interaction between the different modalities, textual information features are also input into the guided attention module under the guidance of the text-complementary prompt module. This further guides the localization of the visual region information, enabling the model to generate more accurate textual and grounded localized visual answers. Subsequently, the visual and textual data refined through layered reasoning are amalgamated using a sophisticated stacked attention mechanism. These combined data are then cohesively encoded alongside visual and textual feature prompts using a data-efficient image transformer (DeiT) [24] encoding. The model was trained using a hybrid loss function that combines cross-entropy loss, L1-norm, and generalized intersection loss, to ensure a comprehensive learning process. Ultimately, this framework culminates in the generation of the final textual and visual referential answers achieved through a dedicated classification head and an object detection head, respectively.

### VCP

This paper proposes a method of visual complementarity to enhance multimodal visually related information for a more accurate inference of ground truth answers through more effective utilization of visual prompting knowledge.

First, as shown in Fig. 1b, both the box prompt features obtained from the pretrained model and visual features encoded through the image encoder are independently encoded using the self-attention mechanism to obtain the prompt features *B*_*s*_ and image encoding *V*_*s*_, thereby enhancing the internal relationships between each feature. The encoded box prompt features are then downsampled to restrict the range of relevant information. Next, the image features *V*_*s*_ are applied using spatial softmax, performing smoothing across all spatial dimensions and employing channel-wise spatial attention to generate the enhanced embedded image features *V*_*m*_ based on the following formula:

1 $${V}_{m}={V}_{s}\odot \left\{\frac{exp\left({V}_{s}^{\left[:,i,j\right]}\right)}{\sum exp\left({V}_{s}^{\left[:,i,j\right]}\right)}\uplambda \right\}$$

Second, the prompt features *B*_*s*_ after downsampling, are effectively integrated with the visual features *V*_*m*_. Drawing inspiration from the segment anything model, two cross-attention fusion modules are used to amalgamate the enhanced visual information features with prompt features. The feature *V*_*c*_ is obtained through cross-attention from the prompt (as a query) directed to the embedded image. In contrast, prompt features *B*_*c*_ are obtained through cross-attention from the embedded image (as a query) directed to the prompts. These two cross-attention mechanisms facilitate the learning of the dependencies between prompt knowledge and visual features. The formulae are as follows:

2 $${B}_{c}= softmax\left( \frac{ {V }_{m} {B}_{d} {}^{T} }{\sqrt{{d}_{k} } } \right){B}_{d}$$

3 $${Y}_{i}=softmax\left( \frac{{B}_{d}{V}_{m} {}^{T} }{\sqrt{{d}_{k} } } \right) {V }_{m}$$

where *T* represents the matrix transpose operation, and *B*_*d*_ is obtained after downsampling *B*_*s*_.

Third, to better guide the localization of the correct answer position with prompt and textual information, visual features *M*_*i*_ are obtained by passing *B*_*c*_ through a residual structure and text-guided attention mechanism [15].

Finally, an iterative fusion strategy is adopted to repeat the process described above, integrating valuable insights by adjusting the prompt and visual information of the instances. Thus, a wealth of details relevant to the domains of both visual and textual features are retained throughout the dynamic process of information exchange. The visual features *O*_*i*_ and prompt features *Y*_*i*_ are then used as new adjusted visual features and box prompt features, which are continuously input into the VCP for iterative updates. This yields the final prompt features and visual features *Y*_6_. The formula is as follows:

4 $${O}_{i}={V}_{m}\oplus {B}_{c}$$

5 $$\langle {O}_{i+1},{Y}_{i+1}\rangle =\Psi \langle {O}_{i},{Y}_{i}\rangle \left(i\le 5\right)$$

where Ψ represents the operation of the VCP presented above, and *i* represents the number of iterations.

### TCP

To effectively extract information from the comparison of features and label prompts across different modalities, this study introduces a text-complementary prompter. As illustrated in Fig. 1c, the label prompt features are initially encoded alongside text information features using a self-attention mechanism, resulting in features denoted as *L*_*s*_ (for label prompts) and *Q*_*s*_ (for text information). Subsequently, label-prompt features *L*_*s*_ undergo upsampling. Drawing inspiration from AFF [23], the positional information of the visual input is projected into two joint feature spaces. This projection is performed alongside the labeling of prompt features and text information using the AFF module, thereby aligning multimodal knowledge and enhancing the interaction of information. The specific formula is as follows:

6 $${Q}_{a}={\Gamma }_{AFF}\left[{Q}_{s},{V}_{s}\right]$$

7 $${C}_{i}={\Gamma }_{AFF}\left[\beta \left({L}_{s}\right),{Q}_{a}\right]$$

where Γ_*AFF*_ represents the AFF operation; *β* represents the upsampling operation; *V*_*s*_ represents input image features.

To derive more meaningful multimodal knowledge prompts from the visual and textual alignment features, the learned multimodal prompts are incrementally integrated into the text feature space using a residual approach. This process utilizes two cross-attention fusion modules in merging the text and prompt features. The text features *R*_*i*_ are acquired by applying cross attention to the prompts (serving as the query) for text encoding. Conversely, prompt features *G*_*i*_ are obtained by applying cross attention to the text encoding (serving as the query) for the prompts. The formula for this process is as follows:

8 $${R}_{i}= softmax\left( \frac{ {Q}_{a} {Q}_{i} {}^{T} }{\sqrt{{d}_{k} } } \right){Q}_{i}$$

9 $${G}_{i}= softmax\left( \frac{{C }_{i}{L }_{i} {}^{T} }{\sqrt{{d}_{k} } } \right) {L }_{i}$$

where *T* represents the matrix transpose operation; *R*_*i*_ represents the visually guided text features; and *G*_*i*_ represents the multimodal information guided prompt features.

As shown in Fig. 1, to avoid excessive guidance from visual information and maintain balance during the six iterations, visual feature information *V*_*i*_ is added only in the first iteration. In subsequent iterations, the prompt multimodal features *C*_*i*_ replace *V*_*i*_ as the input to the module. Throughout the iterations, features *C*_*i*_ interact repeatedly with features *R*_*i*_ and *G*_*i*_ through cross-attention, aiming to guide textual information based on prompt information. This results in the refined text prompt feature *G*_6_ and text information feature *R*_6_. The formula is as follows:

10 $$\langle {C}_{i+1},{R}_{i+1},{G}_{i+1}\rangle =\Phi \langle {C}_{i},{R}_{i},{G}_{i}\rangle \left(i\le 5\right)$$

where *i* is an integer that represents the number of iterations, and Φ represents the text complementary prompt module. To infer instance boundaries and answers relevant to the question from text features *R*_*i*_ and visual features *M*_*i*_ guided by prompt knowledge, we devised a cross-modal feature integration mechanism. This involves the integration of *R*_6_ and *M*_6_ through a fusion module composed of two iAFF modules [23], resulting in the generation of a fused embedding *F*_*i*_:

11 $${F}_{i}={\Gamma }_{iAFF}\left\{{M}_{6},{\Gamma }_{iAFF}\left[{M}_{6},{R}_{6}\right]\right\}$$

The fused feature *F*_*i*_ is input into the pretrained DeiT-base [24] module, and through residual connections, *F*_*i*_ is merged with *Y*_6_ and *G*_6_, further refining the relationships between the features within each domain. Finally, the classification from DeiT is run on the feedforward network, to predict instance bounding boxes and answers relevant to the question.

## Results

### Datasets and evaluation metrics

Experiments were conducted on the EndoVis-2018 [25] and EndoVis-2017 [26] datasets. The EndoVis-2018 comprises video sequences from 14 robotic surgeries [27], with the training set consisting of 1560 frames and 9014 question–answer pairs and the test set comprising 447 frames and 2769 question–answer pairs. The question–answer pairs cover 18 answer categories encompassing various single-word answers related to organs, surgical instruments, and interactions between instruments and organs. For questions involving the interaction between organs and instruments, the bounding box incorporates both the organ and instrument. Each example video contains multiple question–answer pairs along with their corresponding bounding box annotations [14]. The EndoVis-2017 dataset includes video sequences from 10 robotic surgeries with 97 frames containing 472 QA pairs. This dataset was utilized solely for external validation and not as part of the training set.

PSI-AVA dataset, specifically designed for robot-assisted radical prostatectomy surgeries, significantly contributes to the field of surgical scene understanding. PSI-AVA-VQA is an innovative dataset featuring QA pairs derived from critical surgical instances across eight cases from a comprehensive PSI-AVA surgical scene collection. These QA pairs were carefully created from annotations related to surgical phases, steps, and locations within the PSI-AVA collection. With 10,291 QA pairs, the PSI-AVA-VQA dataset encompasses 35 distinct answer categories, including four locations, 11 phases of surgery, and 20 distinct surgical steps. Annotations categorize the QA pairs into three groups: location, phase, and step, adhering to the original PSI-AVA dataset’s fold-1 training/test division methodology.

The reasoning performance of the model was evaluated using the ACC of the text answers and precision of the grounded answer. For grounding answer evaluation, the similarity between each bounding box annotation and ground truth was measured using Intersection over Union (IoU), computing the mean IoU (mIoU) scores for all the test examples. The textual answers were evaluated using two metrics: ACC and F-score.

### Implementation details

The proposed model was trained under cross-entropy loss, L1-norm, and generalized IoU loss using the Adam [28] optimizer, with initial learning rates of 1e-5 for all parameters. The proposed model was trained on the EndoVis-18 training set, with the performance evaluated on the EndoVis-18 validation set, using EndoVis-17 as an external validation dataset to test the model’s generalization ability. The experiments were conducted using the Python PyTorch framework on a server equipped with an NVIDIA Tesla A100 GPU.

### Comparison results

The proposed DMPL model was compared with previous studies on the EndoVis-18 [25] and EndoVis-17 [26] datasets, and the results are reported in Table 1, based on the answering and bounding box metrics. The proposed model surpassed previous advanced methods in most scenarios. Specifically, regarding ACC, F-score, and mIoU score, DMPL outperformed CAT-ViL DeiT [15] by 5.01%, 18.16%, and 1.22% on the EndoVis-18 dataset, and 4.66%, 0.95%, and 1.14% on the EndoVis-17 dataset, respectively. The experimental results indicate that the proposed DMPL achieves superior performance in terms of VQA while maintaining clinical alignment between the answer and related visual instances. This improvement in performance is mainly due to the incorporation of visual and textual supplementary prompts through the two proposed prompters, which assist the model in filtering valid multidomain information.

**Table 1:** Evaluations of different models on EndoVis-18 [25] and EndoVis-17 [26] datasets

Models	VisualFeature		EndoVis-18			EndoVis-17		
	Detection	Inference speed	ACC	F-score	mIoU	ACC	F-score	mIoU
VisualBERT [29]			0.5973	0.3223	0.7340	0.4382	0.3743	0.6822
VisualBERT R [30]			0.6064	0.3226	0.7305	0.4267	0.3506	0.6947
MCAN [31]			0.6084	0.3428	0.7257	0.4258	0.3035	0.6832
VQA-DeiT [24]	FRCNN [32]	55.28 ms	0.6049	0.3238	0.7217	0.4492	0.3213	0.7134
MUTAN [33]			0.6049	0.3238	0.7217	0.4364	0.3206	0.6870
MFH [34]			0.6179	0.3158	0.7227	0.3729	0.2048	**0.7183**
BlockTucker [35]			0.6067	0.3414	0.7313	0.4364	0.3210	0.6825
CAT-ViL DeiT [15]			0.6192	0.3521	0.7482	0.4555	0.3676	0.7049
**DMPL (Ours)**			**0.6461**	**0.4930**	**0.7620**	**0.4760**	**0.3800**	0.7138
VisualBERT [29]			0.6268	0.3329	0.7391	0.4005	0.3381	0.7073
VisualBERT R [30]			0.6301	0.3390	0.7352	0.4190	0.3370	0.7137
MCAN [31]			0.6285	0.3338	0.7526	0.4137	0.2932	0.7029
VQA-DeiT [24]	ResNet18 [36]	6.64 ms	0.6104	0.3156	0.7341	0.3797	0.2858	0.6909
MUTAN [33]			0.6283	0.3395	0.7639	0.4242	0.3482	0.7218
MFH [34]			0.6283	0.3254	0.7592	0.4103	0.3500	0.7216
BlockTucker [35]			0.6201	0.3286	0.7653	0.4221	0.3515	0.7288
CAT-ViL DeiT [15]			0.6452	0.3321	0.7705	0.4491	0.3622	0.7322
**DMPL (Ours)**			**0.6953**	**0.5137**	**0.7827**	**0.4957**	**0.3717**	**0.7436**

**Fig. 2:**
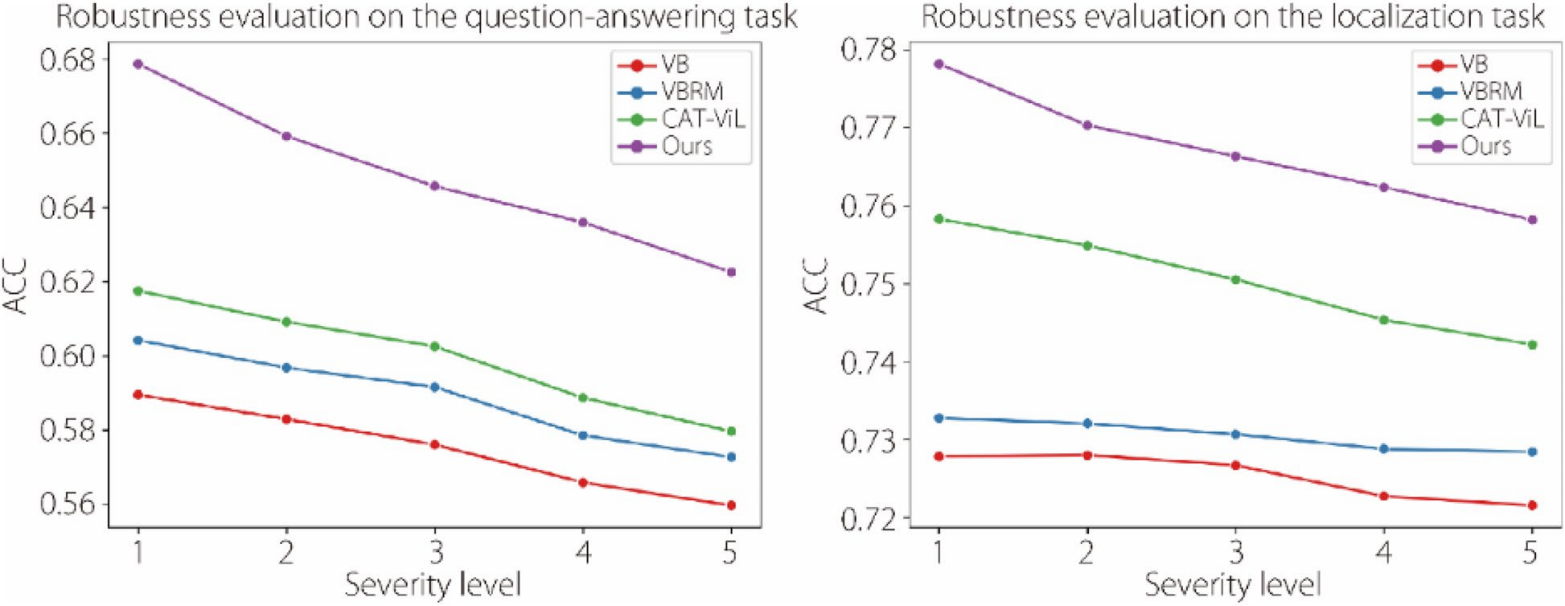
Quantitative robustness experiments on the EndoVis-18 [25] dataset. Experiments were conducted on all types of image corruption at every level of image degradation, and the results were averaged. VB: VisualBERT; VBRM: VisualBERT ResMLP

To further evaluate the robustness of the model, quantitative experiments were conducted to assess performance degradation of the model when confronted with corrupted images. Following the ref. [37], we selected 15 corruption types prevalent in the real world for our experiments and set five levels of corruption for each type. As shown in Fig. 2, the performance of all the models is directly proportional to image quality. However, compared with advanced models, such as VisualBERT [29], VisualBERT ResMLP [30], and CAT-ViL DeiT [15], the proposed DMPL achieves the best performance across all levels of image corruption. This indicates that the proposed model has the capability to maintain robustness when dealing with previously unseen corrupted images, which can be attributed to the prompt knowledge introduced.

To further validate the generalizability of the proposed model, we trained and tested it on the PSI-AVA dataset. As shown in Table 2, compared to the latest models, the proposed model exhibits higher ACC and recall rates on this dataset but lower precision and F-scores. These results suggest that the model can identify most positive samples, indicating a certain level of generalizability. However, many negative samples are misclassified as positive. This issue may arise from the model optimization process not being sufficiently detailed too leniently labeling the samples as positive. Addressing this issue will be the focus of future work.

**Table 2:** Evaluations of different models on the PSI-AVA [38] dataset

Models	PSI-AVA			
	ACC	Precision	Recall	F-score
VisualBERT [29]	0.3008	0.1818	0.6970	0.1408
VisualBERT R [30]	0.2901	0.1927	0.6137	**0.1673**
CAT-ViL DeiT [15]	0.2806	0 1819	0.5967	0.1668
DeepSeek-VL [39]	0.1342	**0.6342**	0.3756	0.0572
ALLaVA [40]	0.1727	0.5829	0.2626	0.1177
**DMPL (Ours)**	**0.3222**	0.1552	**0.7976**	0.1036

## Discussion

### Ablation studies

It would be pertinent to explore the efficacy of each prompter within the proposed model. Thus, an ablation study was conducted, focusing on three configurations: incorporating VCP alone, TCP alone, and a combination of both. Experiments were performed on the EndoVis-18 [25] and EndoVis-17 [26] datasets, maintaining settings consistent with the quantitative outcomes outlined in Table 3.

**Table 3:** Experimental results of ablation studies on EndoVis-18 [25] and EndoVis-17 [26] datasets

Models	EndoVis-18			EndoVis-17		
	ACC	F-score	mIoU	ACC	F-score	mIoU
Co-Attn DeiT [24]	0.6136	0.3208	0.7273	0.3805	0.3026	0.6870
CAT-ViL DeiT [15]	0.6452	0.3321	0.7705	0.4491	0.3622	0.7322
GVLE-LViT [14]	0.6659	0.3614	0.7625	0.4576	0.2489	0.7275
**TCP (Ours)**	0.6845	0.4846	0.7762	0.4639	0.3334	0.7509
**VCP (Ours)**	0.6581	0.4078	0.7740	0.4915	0.3636	**0.7685**
**DMPL (Ours)**	**0.6953**	**0.5137**	**0.7827**	**0.4957**	**0.3717**	0.7436

The results unequivocally illustrate that incorporating either VCP or TCP, individually or concurrently, significantly improves the predictive ACC of the model for both bounding boxes and responses. This surpasses the performance of advanced models across various benchmarks and underscores the essential role played by each prompt in enhancing the proficiency of the model. However, the simultaneous integration of both prompters resulted in a comparatively marginal increase in bounding box prediction ACC compared with independent integration. This observation stems from the methodology of the proposed model in which each prompter contributes complex semantic features sequentially, leading to the transformation of the initial prompter’s contribution into more rudimentary semantic features. This sequential integration may inadvertently affect bounding box prediction ACC by potentially introducing confusion between the complex semantic inputs from the secondary prompter.

**Fig. 3:**
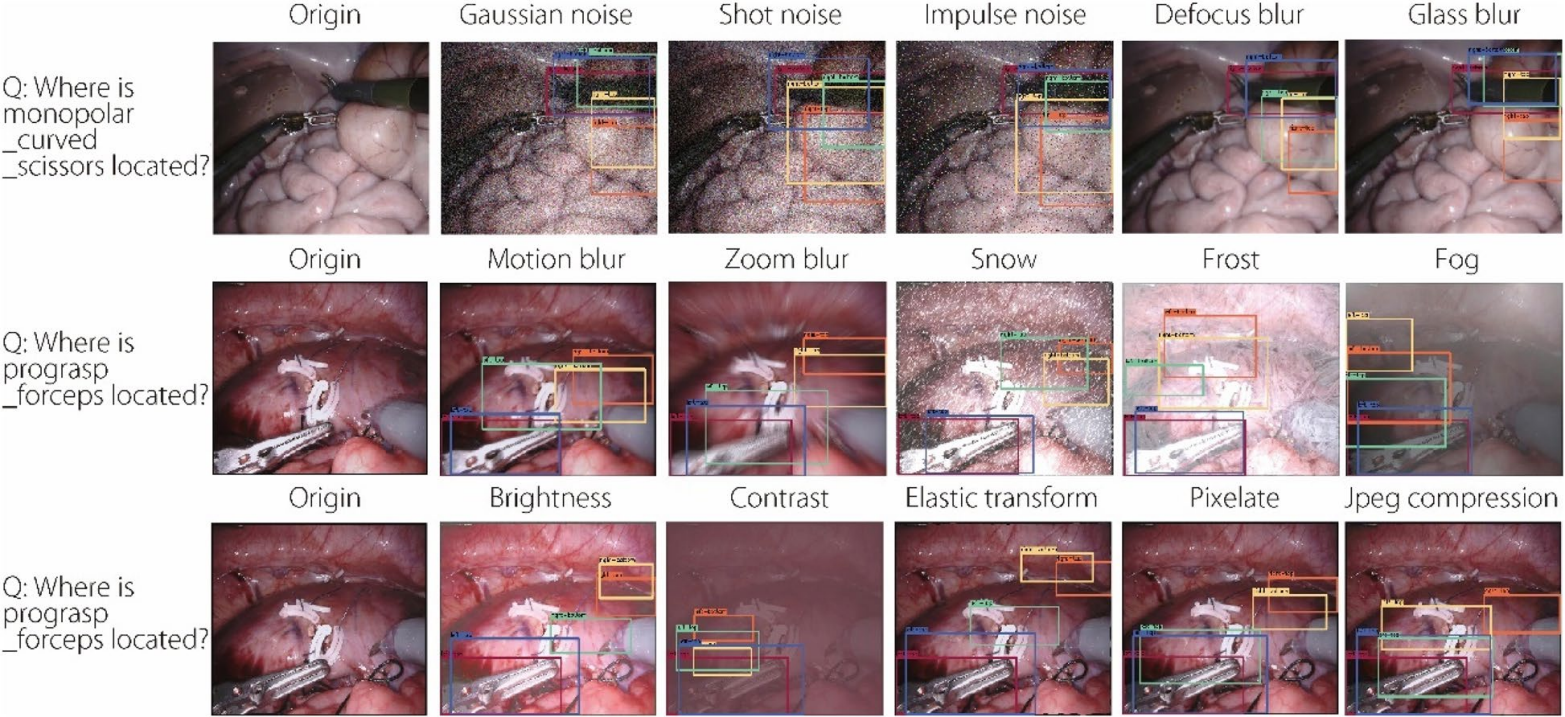
Qualitative robustness experiments on the EndoVis-18 dataset. Experiments were conducted on 15 types of image corruption at level 2 of image degradation to visualize the answers predicted by the models and the associated bounding boxes. The 15 types of image corruption included Gaussian, shot, and impulse noise; defocus, glass, motion, and zoom blur; snow, frost, fog, brightness, contrast, elastic transform, pixelate, and jpeg compression

To further demonstrate the robustness of the proposed model, the qualitative results of VisualBERT [29], VisualBERT ResMLP [30], CAT-ViL DeiT [15], and our model were visualized on the EndoVis-18 dataset [25] for 15 types of image corruptions at level 2 of image degradation. As shown in Fig. 3, various types of image corruption interfere with the localization of bounding boxes in advanced models, thereby indirectly affecting the predictions of answers. By contrast, the proposed model can successfully suppress the interference introduced by image corruption, correctly predicting the answers.

### Qualitative analysis

**Fig. 4:**
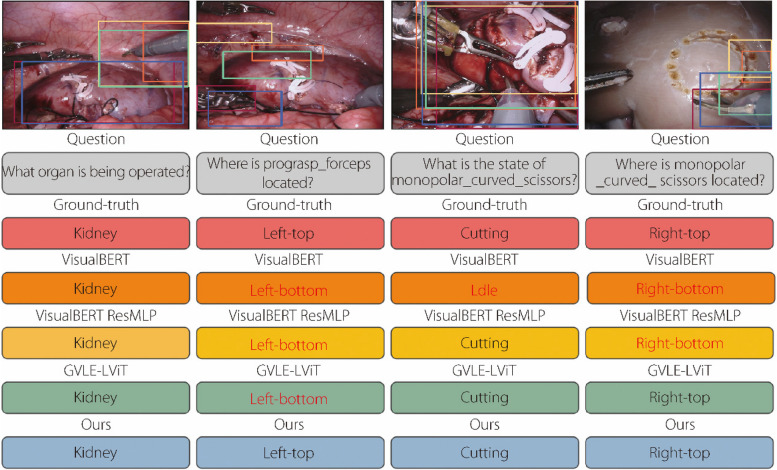
Examples of localization and classification prediction results generated by the proposed model and other advanced models on the EndoVis-18 [25] dataset. Text in red denotes the wrong answer. Examples 1, 2, 3, 4 refer to these four examples from left to right

In Fig. 4, the four sets of sample image-question pairs can be visualized along with the ground-truth answers and generated answers. The proposed model demonstrates a pronounced ability to pinpoint instance locations pertinent to posed questions, markedly enhancing the caliber of the generated responses. For instance, in Example 2, advanced models such as VisualBERT [29], VisualBERT ResMLP [30], and CAT-ViL DeiT [14] erroneously ground the bounding box to the image’s bottom-left corner, which leads to an inaccurate prediction of “bottom-left” as the answer. Conversely, the proposed model accurately identifies the bounding box at the top-left position, providing the correct answer. An analogous outcome is observed in Example 4. The research findings indicate that the VCP framework, through the integration of visual and textual prompt knowledge, effectively disregards irrelevant areas within images. This approach significantly minimizes the distractions in answer prediction, thereby enhancing the precision and focus of the response mechanism.

In Example 1, advanced models excel in predicting accurate answers but struggle to precisely localize relevant bounding boxes. Conversely, VisualBERT [29] in Example 3 adeptly identifies the positions of instances related to the query but fails to deliver the correct answer. This highlights the ongoing challenge for advanced models to seamlessly integrate visual text and location-answer alignments. By contrast, the proposed model consistently achieves these alignments across both examples. This proficiency is attributed to the effective alignment and interaction of multi-domain knowledge within the TCP, mitigating biases linked to dependence on knowledge from isolated domains.

As the EndoVis-17 dataset does not provide training data and is only used for testing, the experimental results from this dataset reflect the generalizability of the model. Therefore, we focused on analyzing the model’s performance on this dataset to acquire a deeper understanding of its strengths and weaknesses.

**Fig. 5:**
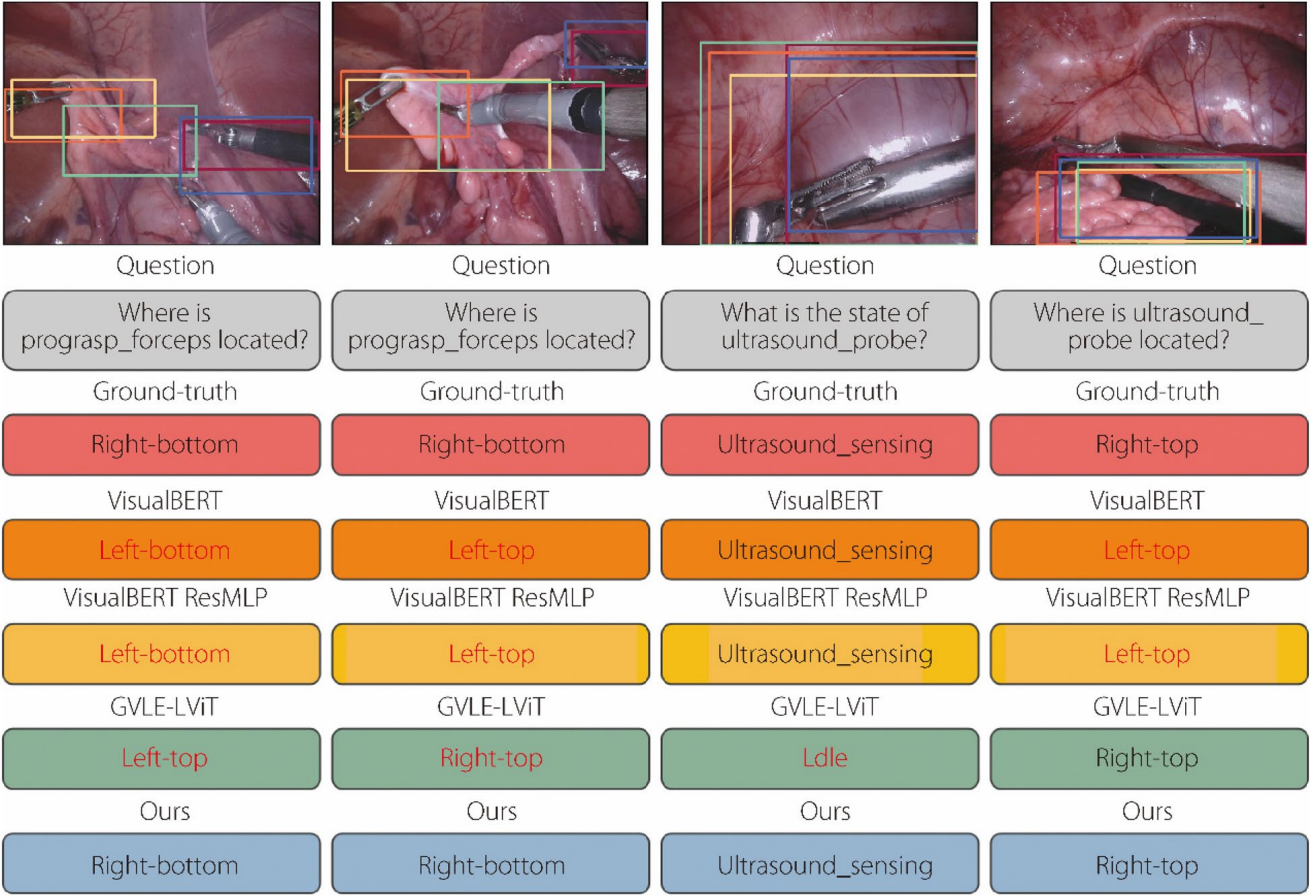
Examples of the true results generated by the proposed model and other models on the EndoVis17 [25] dataset. Text in red denotes wrong answers. Examples 1, 2, 3, 4 refer to these four examples from left to right

As shown in Fig. 5, Examples 1 and 2 demonstrate that the proposed model achieves more accurate visual answer localization, leading to correct textual answers unlike other models generating erroneous answers because of incorrect localization. In Example 3, other models also locate the correct answer, but with an overly broad and imprecise range. This inclusion of excessively irrelevant information results in incorrect textual answers.

These three examples prove that the proposed model significantly improves the precision of visual answer localization compared to previous methods. Example 4 shows that all models identified the correct visual information; however, the answers generated by the other models were still incorrect. This proves that the proposed model, guided by prompt information, is engaged in a more thorough visual and textual information interaction and alignment. These examples demonstrate that the proposed model outperforms previous models in terms of both localization and answer ACC.

### Limitations

The proposed model leverages the advantages of dual-modal prompt learning to some extent to improve the ACC of answers. However, achieving precise semantic alignment between modalities at a fine-grained level still poses certain challenges. In other words, there may be instances where the word in the question does not align accurately with the object in the image.

**Fig. 6:**
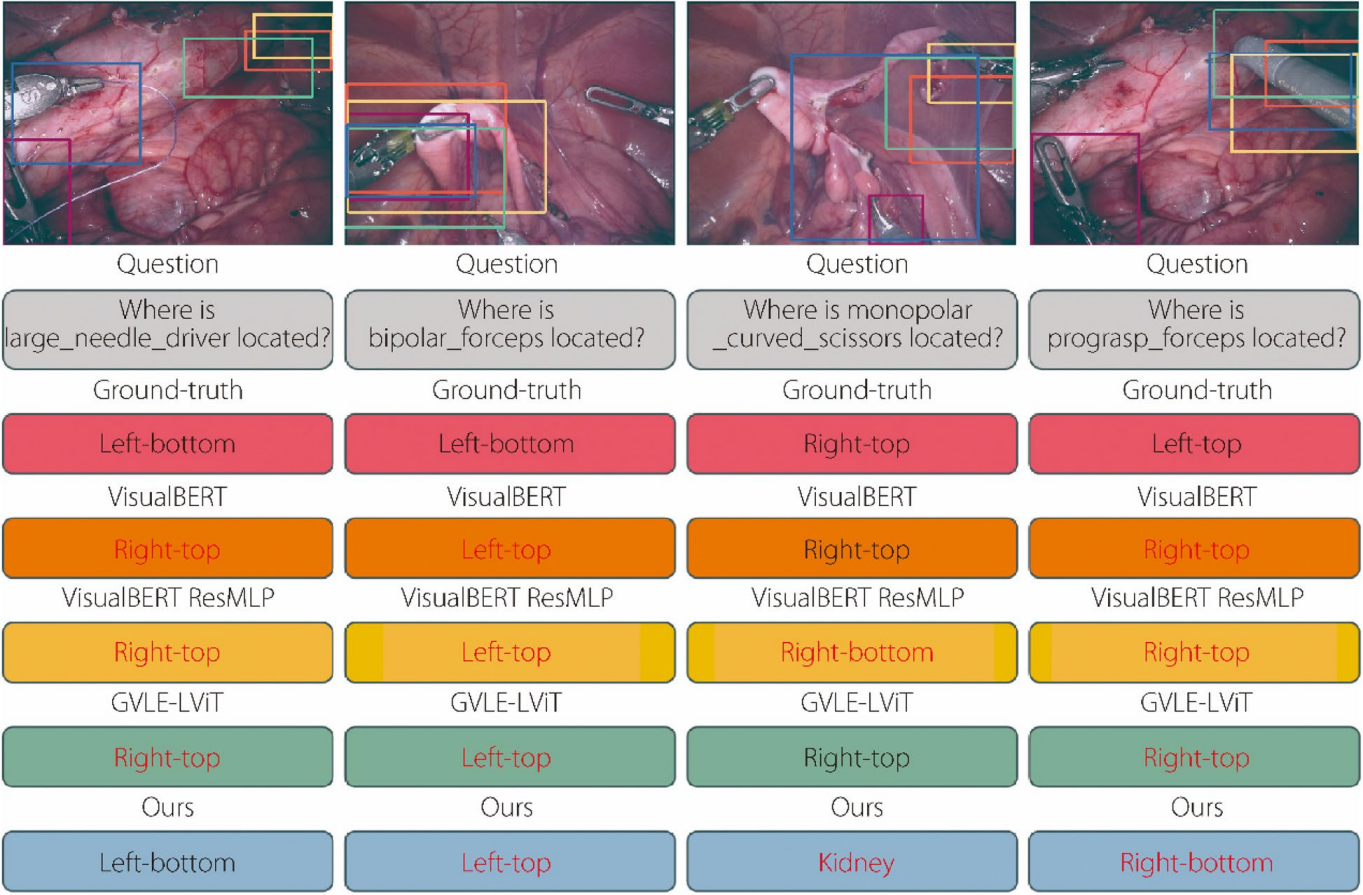
Examples of the proposed and other models failing on the EndoVis-17 [25] dataset. Text in red denotes the wrong answer. Examples 1, 2, 3, 4 denote these four examples from left to right

Figure 6 presents four examples of incorrect results. In Example 1, although the answer is correct, the positioning is incorrect. In Example 2, the positioning is correct but the answer is incorrect. These two examples indicate that the textual and visual information are not sufficiently aligned, leading to discrepancies between the visual and textual answers. Although the proposed approach mitigates language bias to some extent, it remains susceptible to biases inherent in the training data. This can influence the system’s decision-making process, particularly in scenarios in which textual and visual prompts suggest conflicting interpretations.

Example 3 demonstrates an area of localization that is not sufficiently specific, focusing on excessive incorrect visual information, resulting in an incorrect answer. This example also suggests that the model ACC in localizing specific regions, particularly small targets, requires improvement. There is room for the model to enhance its focus on local information. Example 4 shows incorrect localization and answers, indicating that the ACC of the model requires further improvement. Additionally, the volume of training data in the dataset was a limiting factor for the model. The model had to learn from additional data to further improve its ACC.

## Conclusions

In this study, a dual-modality prompt learning framework was designed for VQGA in robotic surgery. The proposed framework leverages the prompt knowledge generated by pretrained models to facilitate the joint encoding of cross-modal inputs, thereby improving the understanding of surgical scenes while localizing specific areas relevant to answering questions. The experimental evaluations conducted on the EndoVis-18 and EndoVis-17 datasets revealed that the proposed model effectively focuses on pertinent regions within images while capturing efficient multimodal alignments. Consequently, the extension of prompt learning into the realm of VQA not only proves beneficial, but also emerges as a promising avenue for future research.

## Data Availability

The data supporting the findings of this study are available from the corresponding author upon request.

## Abbreviations

VQA: Visual question answering
VQGA: Visual question-grounded answering
AFF: Attentional feature fusion
iAFF: Iterative attentional feature fusion
VCP: Visual complementary prompter
TCP: Text complementary prompter
DMPL: Dual modality prompt learning
CAT-ViL: Co-attention gated vision-language embedding
VQLA: Visual question localized-answering
DeiT: Data-efficient image transformer
IoU: Intersection over Union
mIoU: Mean Intersection over Union
ACC: Accuracy

## References

[1] Wu DC, Wang YH, Ma HM, Ai LY, Yang JL, Zhang SJ et al (2023) Adaptive feature extraction method for capsule endoscopy images. Vis Comput Ind Biomed Art 6(1):24. 10.1186/S42492-023-00151-6 38072844 10.1186/s42492-023-00151-6 PMC10710972

[2] Pan J, Lv RJ, Wang Q, Zhao XB, Liu JG, Ai L (2023) Discrimination between leucine-rich glioma-inactivated 1 antibody encephalitis and gamma-aminobutyric acid B receptor antibody encephalitis based on ResNet18. Vis Comput Ind Biomed Art 6(1):17. 10.1186/S42492-023-00144-5 37592180 10.1186/s42492-023-00144-5 PMC10435436

[3] Sarmah M, Neelima A, Singh HR (2023) Survey of methods and principles in three-dimensional reconstruction from two-dimensional medical images. Vis Comput Ind Biomed Art 6(1):15. 10.1186/S42492-023-00142-7 37495817 10.1186/s42492-023-00142-7 PMC10371974

[4] Khan AU, Kuehne H, Duarte K, Gan C, Lobo N, Shah M (2021) Found a reason for me? Weakly-supervised grounded visual question answering using capsules. In: Proceedings of the IEEE/CVF conference on computer vision and pattern recognition. IEEE, Nashville. 10.1109/CVPR46437.2021.00836

[5] Anderson P, He XD, Buehler C, Teney D, Johnson M, Gould S et al (2018) Bottom-up and top-down attention for image captioning and visual question answering. In: Proceedings of the 2018 IEEE/CVF conference on computer vision and pattern recognition. IEEE, Salt Lake City. 10.1109/CVPR.2018.00636

[6] Urooj A, Mazaheri A, Da Vitoria Lobo N, Shah M (2020) MMFT-BERT: multimodal fusion transformer with BERT encodings for visual question answering. In: Proceedings of the association for computational linguistics: EMNLP 2020, Online Event, Association for Computational Linguistics. 10.18653/V1/2020.FINDINGS-EMNLP.417

[7] Hu RH, Rohrbach A, Darrell T, Saenko K (2019) Language-conditioned graph networks for relational reasoning. In: Proceedings of the 2019 IEEE/CVF international conference on computer vision. IEEE, Seoul. 10.1109/ICCV.2019.01039

[8] Jiang Y, Natarajan V, Chen XL, Rohrbach M, Batra D, Parikh D (2018) Pythia v0.1: the winning entry to the VQA challenge 2018. arXiv preprint arXiv:1807.09956

[9] Zhou Y, Ren T, Zhu C, Sun X, Liu J, Ding X et al (2021) TRAR: Routing the Attention Spans in Transformer for Visual Question Answering. In: Proceedings of 2021 IEEE/CVF International Conference on Computer Vision. IEEE, Montreal. 10.1109/ICCV48922.2021.00208

[10] Reich D, Putze F, Schultz T (2023) Measuring Faithful and Plausible Visual Grounding in VQA. In: Findings of the Association for Computational Linguistics: EMNLP 2023, Association for Computational Linguistics, Singapore, pp 3129–3144. 10.18653/v1/2023.findings-emnlp.206

[11] Gan C, Li YD, Li HX, Sun C, Gong BQ (2017) VQS: linking segmentations to questions and answers for supervised attention in VQA and question-focused semantic segmentation. In: Proceedings of the IEEE international conference on computer vision. IEEE, Venice. 10.1109/ICCV.2017.201

[12] Hudson DA, Manning CD (2019) GQA: a new dataset for real-world visual reasoning and compositional question answering. In: Proceedings of the IEEE/CVF conference on computer vision and pattern recognition. IEEE, Long Beach. 10.1109/CVPR.2019.00686

[13] Chen CY, Anjum S, Gurari D (2022) Grounding answers for visual questions asked by visually impaired people. In: Proceedings of the IEEE/CVF conference on computer vision and pattern recognition. IEEE, New Orleans. 10.1109/CVPR52688.2022.01851

[14] Bai L, Islam M, Seenivasan L, Ren HL (2023) Surgical-VQLA: transformer with gated vision-language embedding for visual question localized-answering in robotic surgery. In: Proceedings of the IEEE international conference on robotics and automation, IEEE, London, 29 May-2 June 2023. 10.1109/ICRA48891.2023.10160403

[15] Bai L, Islam M, Ren HL (2023) CAT-ViL: co-attention gated vision-language embedding for visual question localized-answering in robotic surgery. In: Greenspan H, Madabhushi A, Mousavi P, Salcudean S, Duncan J, Syeda-Mahmood T et al (eds) Medical image computing and computer assisted intervention MICCAI 2023. 26th international conference, Vancouver, October 2023. Lecture notes in computer science, vol 14228. Springer, Cham, pp 397–407. 10.1007/978-3-031-43996-4_38

[16] Bai L, Islam M, Ren HL (2023) Revisiting distillation for continual learning on visual question localized-answering in robotic surgery. In: Greenspan H, Madabhushi A, Mousavi P, Salcudean S, Duncan J, Syeda-Mahmood T, et al (eds) Medical image computing and computer assisted intervention MICCAI 2023. 26th international conference, Vancouver, October 2023. Lecture notes in computer science, vol 14228. Springer, Cham, pp 68–78. 10.1007/978-3-031-43996-4_7

[17] Tascon-Morales S, Márquez-Neila P, Sznitman R (2023) Localized questions in medical visual question answering. In: Greenspan H, Madabhushi A, Mousavi P, Salcudean S, Duncan J, Syeda-Mahmood T et al (eds) Medical image computing and computer assisted intervention MICCAI 2023. 26th international conference, Vancouver, October 2023. Lecture notes in computer science, vol 14221. Springer, Cham, pp 361–370. 10.1007/978-3-031-43895-0_34

[18] Lester B, Al-Rfou R, Constant N (2021) The power of scale for parameter-efficient prompt tuning. In: Proceedings of the 2021 conference on empirical methods in natural language processing. Association for Computational Linguistics, Punta Cana. 10.18653/V1/2021.EMNLP-MAIN.243

[19] Liu PF, Yuan WZ, Fu JL, Jiang ZB, Hayashi H, Neubig G (2023) Pre-train, prompt, and predict: a systematic survey of prompting methods in natural language processing. ACM Comput Surv 55(9):195.10.1145/3560815

[20] Jia ML, Tang LM, Chen BC, Cardie C, Belongie S, Hariharan B et al (2022) Visual prompt tuning. In: Avidan S, Brostow G, Cissé M, Farinella GM, Hassner T (eds) Computer vision - ECCV 2022. 17th European conference, Tel Aviv, October 2022. Lecture notes in computer science, vol 13693. Springer, Cham, pp 709–727. 10.1007/978-3-031-19827-4_41

[21] Chen SF, Ge CJ, Tong Z, Wang JL, Song YB, Wang J et al (2022) AdaptFormer: adapting vision transformers for scalable visual recognition. In: Proceedings of the 36th conference on neural information processing systems, New Orleans

[22] Jie SB, Deng ZH (2022) Convolutional bypasses are better vision transformer adapters. arXiv preprint arXiv: 2207.07039. 10.48550/ARXIV.2207.07039

[23] Dai YM, Gieseke F, Oehmcke S, Wu YQ, Barnard K (2021) Attentional feature fusion. In: Proceedings of the IEEE winter conference on applications of computer vision, IEEE, Waikoloa. 10.1109/WACV48630.2021.00360

[24] Touvron H, Cord M, Douze M, Massa F, Sablayrolles A, Jégou H (2021) Training data-efficient image transformers & distillation through attention. In: Proceedings of the 38th international conference on machine learning, ICML, Virtual Event

[25] Allan M, Kondo S, Bodenstedt S, Leger S, Kadkhodamohammadi R, Luengo I et al (2020) 2018 Robotic scene segmentation challenge. arXiv preprint arXiv: 2001.11190

[26] Allan M, Shvets A, Kurmann T, Zhang ZC, Duggal R, Su YH et al (2019) 2017 Robotic instrument segmentation challenge. arXiv preprint arXiv: 1902.06426

[27] Seenivasan L, Mitheran S, Islam M, Ren HL (2022) Global-reasoned multi-task learning model for surgical scene understanding. IEEE Robot Autom Lett 7(2):3858–3865. 10.1109/LRA.2022.3146544

[28] Kingma DP, Ba J (2015) Adam: a method for stochastic optimization. In: Proceedings of the 3rd international conference on learning representations, San Diego

[29] Li LH, Yatskar M, Yin D, Hsieh CJ, Chang KW (2019) VisualBERT: a simple and performant baseline for vision and language. arXiv preprint arXiv: 1908.03557

[30] Seenivasan L, Islam M, Krishna AK, Ren HL (2022) Surgical-VQA: visual question answering in surgical scenes using transformer. In: Wang LW, Dou Q, Fletcher PT, Speidel S, Li S (eds) Medical image computing and computer assisted intervention – MICCAI 2022. 25th international conference, Singapore, September 2022. Lecture notes in computer science, vol 13437. Springer, Cham, pp 33–43. 10.1007/978-3-031-16449-1_4

[31] Yu Z, Yu J, Cui YH, Tao DC, Tian Q (2019) Deep modular co-attention networks for visual question answering. In: Proceedings of the IEEE/CVF conference on computer vision and pattern recognition, IEEE, Long Beach. 10.1109/CVPR.2019.00644

[32] Ren SQ, He KM, Girshick R, Sun J (2017) Faster R-CNN: towards real-time object detection with region proposal networks. IEEE Trans Pattern Anal Mach Intell 39(6):1137–1149. 10.1109/TPAMI.2016.2577031 27295650 10.1109/TPAMI.2016.2577031

[33] Ben-Younes H, Cadene R, Cord M, Thome N (2017) MUTAN: multimodal tucker fusion for visual question answering. In: Proceedings of the IEEE international conference on computer vision. IEEE, Venice. 10.1109/ICCV.2017.285

[34] Yu Z, Yu J, Xiang CC, Fan JP, Tao DC (2018) Beyond bilinear: generalized multimodal factorized high-order pooling for visual question answering. IEEE Trans Neural Netw Learn Syst 29(12):5947–5959. 10.1109/TNNLS.2018.2817340 29993847 10.1109/TNNLS.2018.2817340

[35] Ben-Younes H, Cadene R, Thome N, Cord M (2019) BLOCK: bilinear superdiagonal fusion for visual question answering and visual relationship detection. In: Proceedings of the 33rd AAAI conference on artificial intelligence, AAAI Press, Honolulu, 27 January-1 February 2019. 10.1609/AAAI.V33I01.33018102

[36] He KM, Zhang XY, Ren SQ, Sun J (2016) Deep residual learning for image recognition. In: Proceedings of the 2016 IEEE conference on computer vision and pattern recognition. IEEE, Las Vegas. 10.1109/CVPR.2016.90

[37] Hendrycks D, Dietterich TG (2019) Benchmarking neural network robustness to common corruptions and perturbations. In: Proceedings of the 7th international conference on learning representations, OpenReview.net, New Orleans

[38] Valderrama N, Puentes PR, Hernández I, Ayobi N, Verlyck M, Santander J et al (2022) Towards holistic surgical scene understanding. In: Wang LW, Dou Q, Fletcher PT, Speidel S, Li S (eds) Medical image computing and computer assisted intervention – MICCAI 2022. 25th international conference, Singapore, September 2022. Lecture notes in computer science, vol 13437. Springer, Cham, pp 442–452. 10.1007/978-3-031-16449-1_42

[39] Lu HY, Liu W, Zhang B, Wang BX, Dong K, Liu B et al (2024) DeepSeek-VL: towards real-world vision-language understanding. arXiv preprint arXiv: 2403.05525. 10.48550/arXiv.2403.05525

[40] Chen GH, Chen SN, Zhang RF, Chen JY, Wu XB, Zhang ZY et al (2024) ALLaVA: harnessing GPT4V-synthesized data for a lite vision-language model. arXiv preprint arXiv: 2402.11684. 10.48550/ARXIV.2402.11684

